# Hybrid Attention based Multimodal Network for Spoken Language Classification

**Published:** 2018-08

**Authors:** Yue Gu, Kangning Yang, Shiyu Fu, Shuhong Chen, Xinyu Li, Ivan Marsic

**Affiliations:** Multimedia Image Processing Lab, Electrical and Computer Engineering Department, Rutgers University, Piscataway, NJ, USA

## Abstract

We examine the utility of linguistic content and vocal characteristics for multimodal deep learning in human spoken language understanding. We present a deep multimodal network with both feature attention and modality attention to classify utterance-level speech data. The proposed hybrid attention architecture helps the system focus on learning informative representations for both modality-specific feature extraction and model fusion. The experimental results show that our system achieves state-of-the-art or competitive results on three published multimodal datasets. We also demonstrated the effectiveness and generalization of our system on a medical speech dataset from an actual trauma scenario. Furthermore, we provided a detailed comparison and analysis of traditional approaches and deep learning methods on both feature extraction and fusion.

## 1 Introduction

Understanding human conversation is fundamental for human-computer interaction (HCI) and artificial intelligence (AI). However, it is hard for a computer to precisely interpret human meaning because: 1. Giving computers the ability to interpret speech requires a complete understanding of how it works for a human. Unfortunately, we still cannot identify how humans understand the information during conversation. 2. It is hard to extract associated features; there is a gap between the extracted modality-specific features and the actual human state of mind. 3. During conversation, people often accept messages from multiple sources such as facial expression, gesture, linguistic content, and vocal signals. But how to integrate the heterogeneous inputs into a computer is still an open-ended question. In this paper, we focus on exploring and addressing the issues above on the spoken language classification task, which is the most commonly used method for human sentiment analysis, emotion recognition, and speech topic categorization.

Comprehending spoken language can be rephrased as analyzing vocal signals. A variety of research focuses on extracting the human meaning from audio data (Koolagudi et al., 2012; Busso et al., 2013; Mirsamadi et al., 2017). However, instead of directly processing audio information at the frame-level as a computer does, humans comprehend the meaning of utterances on the word-level, which can be seen as extracting meaning from linguistic content. It is natural for beginners in English to translate speech information into their native language word-by-word to understand the conversation, effectively using textual information for spoken language understanding. Based on this assumption, we propose a multimodal structure that considers both the audio signal and text as inputs to classify utterance-level speech data, where text data can be either transcribed speech or automatic speech-to-text output.

Another challenge for classifying spoken language is extracting the associated features. As mentioned in previous research (Poria et al., 2017a), traditional approaches used handcrafted features for both the text and audio branches, which cannot fully represent the high-level characteristics. Recent studies tried to overcome such issues with deep learning structures, including convolutional neural networks (CNNs) (Poria et al., 2015) and long short-term memory networks (LSTMs) (Zadeh et al., 2017). However, the words and audio frames should have different importance. For example, the word hate carries all the anger information in “I hate you”. Even the same word or acoustic frame may have different contributions for different classes. To select the informative words and frames, we introduced an LSTM with an attention mechanism as the feature extractor on both the text and audio branches. A weighted pooling strategy was applied over the feature extractor to form a modality-specific feature representation. Compared to the previous research using independent software or networks to extract the modality-specific features, our system simultaneously tunes both the feature extraction and fusion modules, encouraging optimal feature learning.

Decision-level fusion is a commonly used strategy for fusing heterogeneous inputs, combining the independent modality outputs by using several specific rules. However, the lack of mutual association learning across modalities is a major limitation of applying decision-level fusion (Zhang et al., 2017). Feature-level fusion aims to fuse the extracted modality-specific features as a general feature vector, and so has the ability to learn correlations across modalities. Unfortunately, it is difficult to ensure data synchronization, as different features may consist of diverse time scales and formats (Poria et al., 2017a). To address the above issues, model-level fusion has been introduced, which generates a joint or shared feature representation consisting of both the feature-level fusion and decision-level fusion characteristics. Instead of using shallow structures in fusion as previous approaches did (Kim et al., 2013; Pang et al., 2015), we designed a modality attention fusion that allows system fusion at the feature-level and applies weighted modality scores over the extracted features to indicate the importance of different modalities. This keeps advantages of both feature-level and decision-level fusion.

We evaluated our system on three published multimodal datasets for spoken language understanding tasks, including speech sentiment analysis (CMU-MOSI and MOUD) and speech emotion recognition (IEMOCAP). Our system achieves state-of-the-art on CMU-MOSI and IEMOCAP, and competitive results on MOUD. We also generalize the system to classify speech content for utterance-level data on an an actual trauma resuscitation speech dataset (TRS). The results show that the proposed network improves performance on noisy data. Specifically, this paper addresses the following issues:

1. Does multi-source input data improve performance, and is it necessary to apply text information to a spoken language classification task?
2. Is the proposed attention-based LSTM structure helpful? Does extracting high-level representations perform better than low-level handcrafted features?
3. Is modality attention needed?
4. Is the proposed system enough to perform spoken language classification in a real scenario?

The paper is organized as follows: we present the related work in section 2. Section 3 describes the proposed network. We provide the experiments in section 4 and discuss the results in detail in section 5. We conclude in section 6.

## 2 Related Work

Previous works in spoken language classification focused on extracting acoustic features from different aspects of speech data (Koolagudi et al., 2012; Busso et al., 2013; Mirsamadi et al., 2017). While there is much previous research using audio-visual data on emotion recognition and sentiment analysis, only a few of them consider text as input. These approaches are difficult to generalize to spoken language understanding (due to the lack of visual data in many scenarios) and ignore the contribution of text. Recent studies on speech-based analysis have proposed to use both audio and text data for classification (Jin et al., 2015; Gu et al., 2018). However, they only applied the experiments to emotion recognition.

A variety of feature extraction strategies were proposed in the last decade. Early research used low-level acoustic descriptors and derivations (LLDs) with functional statistics as acoustic features (Rosas et al., 2013). For textual features, they used SVMs with bag of words (BoW) and part of speech (PoS) features in addition to low-level acoustic features (Rozgic et al., 2012; Rosas et al., 2013). Since low-level features represent limited high-level associations (Poria et al., 2015), various deep learning approaches have been proposed, like CNNs (Poria et al., 2016) and LSTMs (Gu et al., 2017b; Zadeh et al., 2017), to learn high-level representations. To further improve system performance, an attention mechanism was introduced in machine translation and text classification (Bahdanau et al., 2014; Yang et al., 2016)

There exist two commonly used fusion strategies in previous research: decision-level fusion and feature-level fusion. Specifically, Poria et al. (Poria et al., 2015, 2016) used a multiple kernel learning strategy to fuse the modality data on the feature-level. A decision-level fusion was applied by Wӧllmer et al. (Wӧllmer et al., 2013) that combines the results of the text and audio-visual modalities by a threshold score vector. Deep neural network fusion was proposed in a recent study to fuse the extracted modality-specific features (Zhang et al., 2017; Gu et al., 2018). More recent approaches introduced LSTM structures to fuse the features at each time step (Poria et al., 2017b; Chen et al., 2017)

## 3 Method

**Figure 1:**
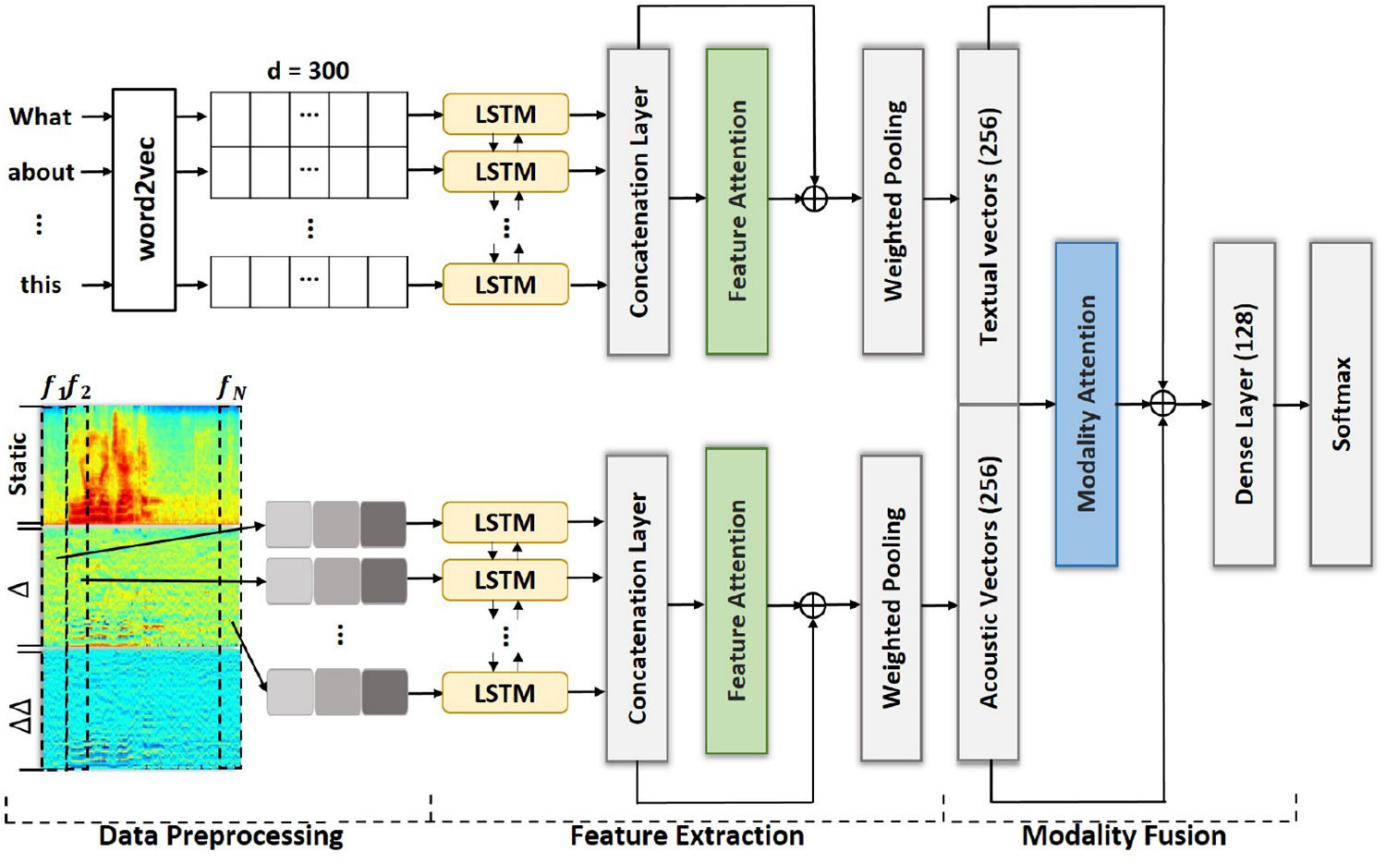
Overall system structure.

We introduce the design of the proposed architecture in this section (shown in Figure 1). There are three major parts of the system: the data preprocessing, feature extraction, and modality fusion.

### 3.1 Data Preprocessing

The system accepts raw audio signal and text as inputs. The data preprocessing module formats the heterogeneous inputs into specific representations, which can be effectively used in the feature extraction network. We embedded the words and extracted Mel-frequency spectral coefficients (MFSCs) from the text and audio inputs for the feature extraction module.

We first embedded each word into a 300-dimensional word vector by *word2vec*, which is a pre-trained word embedding dictionary trained on 100 million words from Google news (Mikolov et al., 2013). Compared to *GloVe* and *LexVec*, *word2vec* provides us the best performance. For all embedded vectors, we allow fine-tuning of the embedding layer via backpropagation during the training stage. We removed all punctuation, as spoken language does not provide tokens. Unknown words were randomly initialized and each sentence was represented as a *N ×* 300 matrix, where *N* is the number of the words for the given sentence.

Unlike most previous research extracting LLDs or using Mel-frequency cepstral coefficients (MFCCs) as the acoustic features (Poria et al., 2016; Mirsamadi et al., 2017), we represented the raw audio signal using MFSCs because: 1. MFSCs maintain the locality of the data by preventing new bases of spectral energies resulting from discrete cosine transform in MFCCs extraction (Abdel-Hamid et al., 2014). 2. Compared to the MFCCs that only have 39 dimensions for each audio frame, MFSCs allow more dimensions in the frequency domain that aid learning in deep models. 3. Instead of using MFCCs, voice intensity, pitch, etc. as in (Poria et al., 2017b) that need voice normalization and statistic computations, MFSC extraction does not require additional operations. As suggested in (Gu et al., 2017), we used 64 filter banks to extract static, delta (Δ), and double delta (ΔΔ) of the MFSCs as the MFSCs map. The final representation is a 3-D array with 64 *× F ×* 3 dimensions, where *F* is number of extracted MFSCs frames.

### 3.2 Textual Feature Extraction with Attention

We applied the LSTM structure with an attention mechanism to extract temporal associations and select informative words.

**Figure 2:**
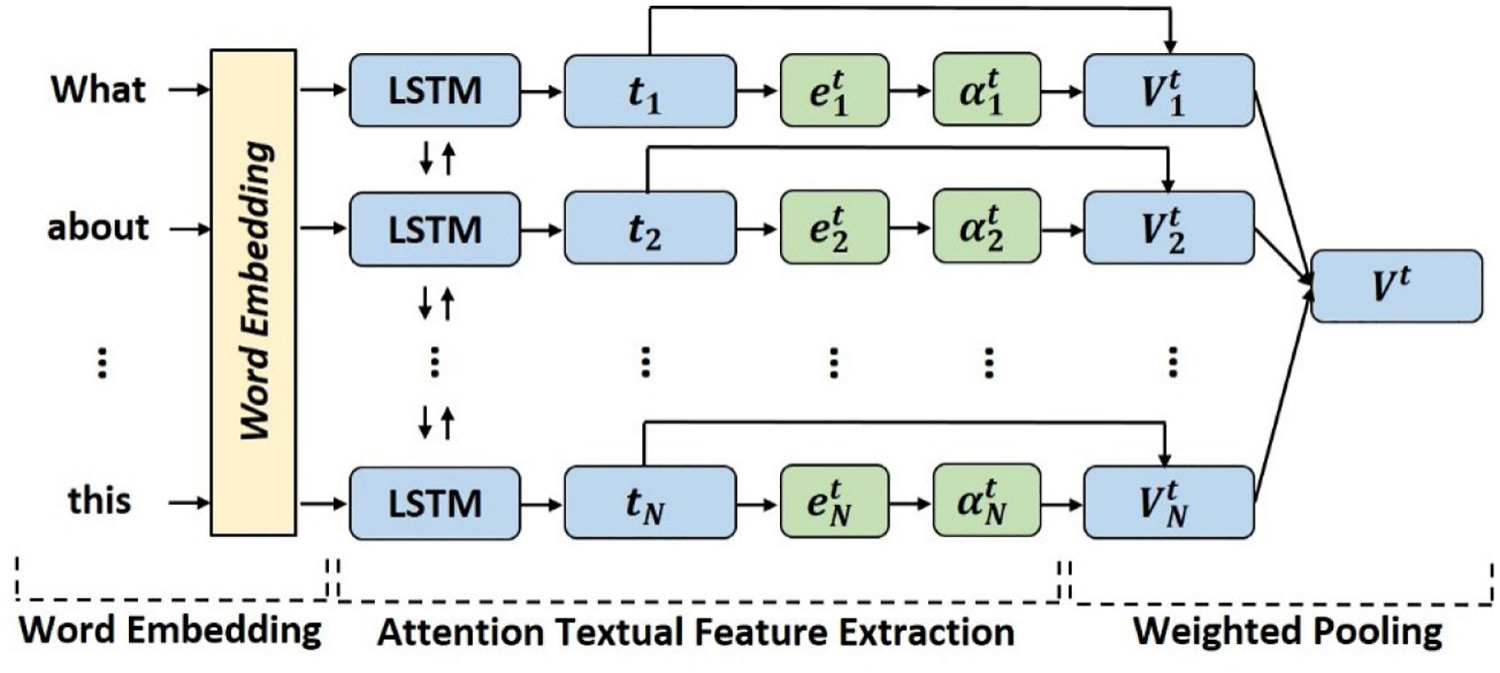
Textual feature extraction with attention.

The textual feature extraction module consists of two parts. Firstly, it has a regular bidirectional LSTM structure used to generate the contextual hidden states for each word vector. Secondly, it has an attention layer connected to the bidirectional LSTM to provide a weight vector over the contextual hidden states to amplify the representative vectors. As shown in Figure 2, we fed the words into the bidirectional LSTM in sequence. Specifically,

$$t_{i}^{\to },t_{i}^{\leftarrow }=bi\_LSTM\left(E_{i}\right),i\in \left[1,N\right]$$

where *E*_*i*_ is the embedded word vector of the *i*th word, *bi_LST M* is the bidirectional LSTM, and $t_{i}^{\to }$ and $t_{i}^{\leftarrow }$ denote respectively the forward and backward contextual states of the given input word vector. Each contextual state is a word-level feature representation with forward and backward temporal associations. As not all words equally contribute to the final prediction, we added a learnable attention layer over the contextual states to denote the importance of the representations. As defined by (Bahdanau et al., 2014), we first computed the text attention energies $e_{i}^{t}$ by:

$$e_{i}^{t}=tanh\left(W_{t}\left[t_{i}^{\to },t_{i}^{\to }\right]+b_{t}\right),i\in \left[1,N\right]$$

Then, we calculated the text attention distribution $\alpha_{i}^{t}$ for word representations via a softmax function:

$$\alpha_{i}^{t}=\frac{exp\left(e_{i}^{t}{}^{⊤}v_{t}\right)}{\sum_{k=1}^{N}exp\left(e_{k}^{t}{}^{⊤}v_{t}\right)}$$

where *W*_*t*_, *b*_*t*_, and *v*_*t*_ are the learnable parameters. To form the final textual feature representation (*V*^*t*^), we applied a weighted-pooling by computing a weighted sum of the text contextual states and the attention distribution:

$$V^{t}=\overset{N}{\underset{i=1}{\sum }}\left[t_{i}^{\to },t_{i}^{\leftarrow }\right]\alpha_{i}^{t}$$

Unlike the systems that apply convolutional neural networks to extract the sentimental and emotional textual features using a fixed window size (Poria et al., 2015; Poria et al., 2017b), we used LSTM structures that can fully capture the sequential information with varying length and learn the temporal associations between words. We notice that Zadeh also applied LSTMs as the textual feature extractor (Zadeh et al., 2017). However, they used a mean-pooling strategy to form the final utterance-level feature representation by passing all the contextual states into the dense layer. This assumes all the outputs can correctly contribute to the final prediction. Unfortunately, as we know, even the same word may carry diverse information that may make a different contributions to the final prediction. The proposed attention layer allows the system to focus on the most informative words to further improve the representations.

### 3.3 Acoustic Feature Extraction with Attention

Similar to textual feature extraction, we also introduced a bidirectional LSTM with attention to focus on extracting informative contextual states on frame-level MFSCs.

Unlike the textual feature extraction that only has one channel (2D-array), the input MFSCs map is a 3D-array. We first concatenated the synchronized frames from static, delta, and double delta feature maps to form the input acoustic feature vector (*A*_*j*_):

$$A_{j}=\left[s_{j},\Delta_{j},\Delta \Delta_{j}\right],j\in \left[1,F\right]$$

Again, we used the same approach as in textual feature extraction to compute the bidirectional acoustic contextual states ($\left[a_{j}^{\to },a_{j}^{\leftarrow }\right]$), acoustic attention energies ($e_{j}^{a}$), and acoustic attention distribution ($\alpha_{j}^{a}$). The ($\alpha_{j}^{a}$) can be understood as the importance score for the jth frame. We computed the weighted sum of the bidirectional acoustic contextual states and acoustic attention distribution as the final acoustic representation (*V*^*a*^).

Unlike previous research that directly uses the acoustic LLDs as the extracted features (Degottex et al., 2014; Poria et al., 2016), the proposed architecture learns high-level acoustic associations. We didn’t use convolutional neural networks to extract the acoustic features as in (Gu et al., 2017) because CNNs only capture spatial associations whereas acoustic data contains many temporal associations. The fixed window size of CNNs limits the temporal interaction extraction. As the number of audio frames is large (hundreds per sentence), the LSTM structure ensures the system captures long-term dependencies among the MFSCs frames. Even if a deep neural network was used for extracting the high-level associations on LLDs (Zadeh et al., 2017; Gu et al., 2018), the generation of attention over the extracted features is still desirable, as it can help indicate the importance at the frame-level. The weighted pooling based on the attention distribution makes sure the final acoustic feature representations contain the most informative features.

### 3.4 Modality Fusion

Simply concatenating the features cannot reveal the actual importance of different modalities; the same modality may have different contributions in different spoken language understanding tasks. For example, people rely more on the vocal delivery and acoustic characteristics to express their emotions, but linguistic content and text are more important to speech content classification. Even for the same task, the modality may have distinct influences on different categories. Acoustic information might provide useful information for the *Anger* class, but it is hard to distinguish *neutral* and *happy* without considering text. To make the system learn this difference, we proposed a modality attention fusion that puts an attention layer over the extracted modality-specific features, helping the system focus on the informative modality. It can be intuitively understood as giving a weighted score vector at the modality-level to indicate the importance of individual branches.

**Figure 4:**
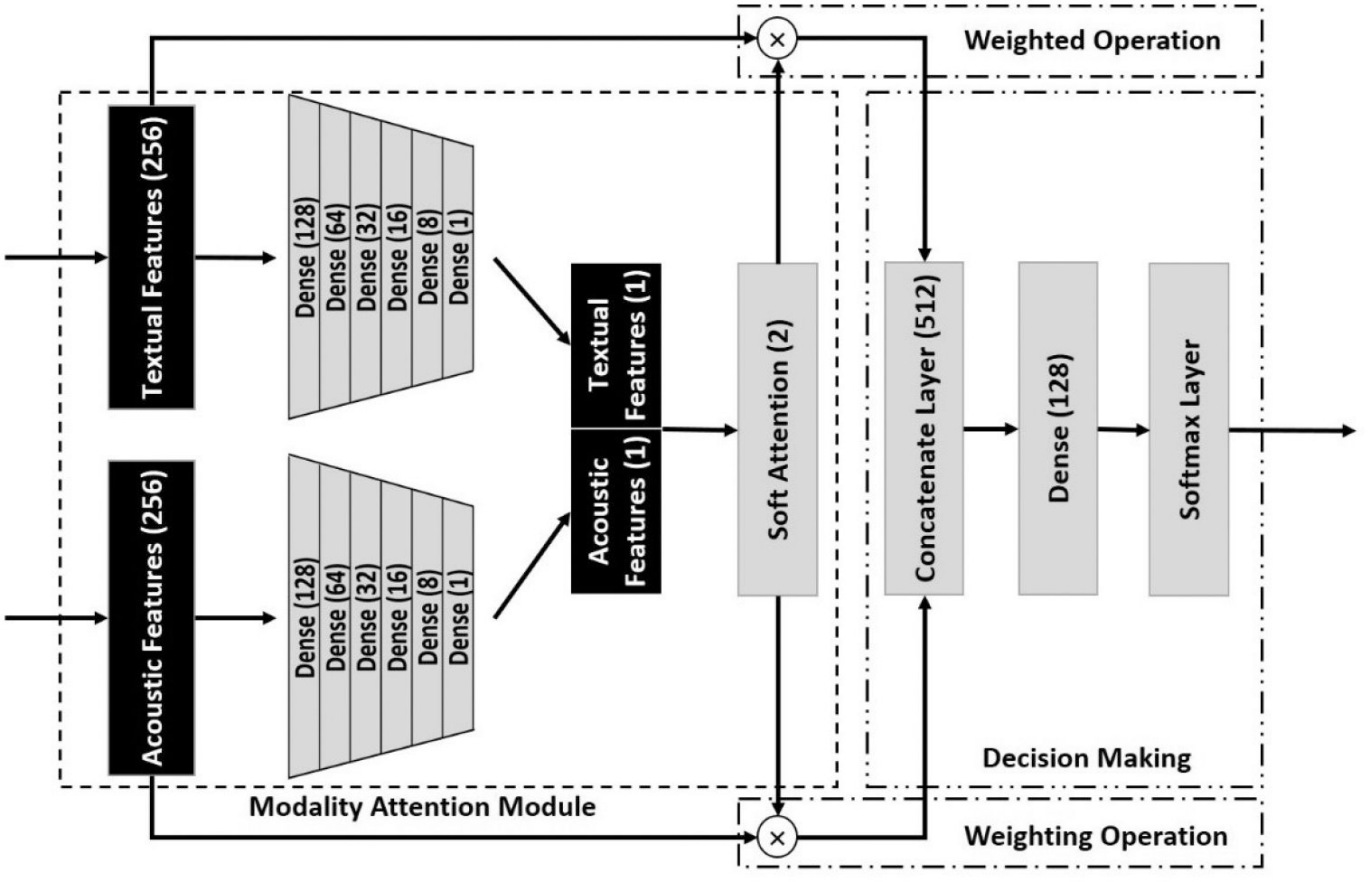
Modality Fusion

The proposed modality fusion consists of three major parts: a modality attention module, a weighted operation, and a decision making module. We first set up five dense layers after the attention layer to fuse the modality-specific features (as shown in figure 4). Then, we used softmax regression to generate the weighted score (*s*) for the given modality:

$$s=softmax\left(tanh\left(W_{f}\left[V_{t}^{*},V_{a}^{*}\right]+b_{f}\right)\right)$$

where *W*_*f*_ and *b*_*f*_ are the trainable fusion attention parameters, *s* is a n-dimension vector, and n=2 in this study (representing the text and audio modalities respectively). We computed a soft-attention over the original modality features and concatenated them. A dense layer was used to learn the associations across weighted modality-specific features by:

$$r=tanh\left(W_{r}\left[\left(1+s_{t}\right)V^{t},\left(1+s_{a}\right)V^{a}\right]+b_{r}\right)$$

where *r* is the final representation, and *W*_*r*_ and *b*_*r*_ are the additional parameters for the last dense layer. We used (1 + *s*) as the attention score to keep the original modality characteristics. We made the final decision by a softmax classifier using r as input.

## 4 Experiment

We evaluated the proposed system on three published multimodal datasets and an actual trauma resuscitation speech dataset. We compared our structure with the baselines from three major aspects: 1. proposed system vs previous methods; 2. low-level handcrafted features vs high-level features; 3. shallow fusion vs deep fusion. We also conducted an experiment on a trauma resuscitation speech dataset that uses speech-to-text results as text input to test the generalizability of the system.

### 4.1 Dataset

We selected three multimodal datasets that contain spoken language information. We used audio and text data as inputs in this study. Table 1 shows dataset details.

**CMU-MOSI:** This dataset is a multimodal sentiment intensity and subjectivity dataset consisting of 93 review videos in English with 2199 utterance segments (Zadeh et al., 2016). Each segment is labelled by five individual annotators between −3 (strong negative) to +3 (strong positive). The aim of using this dataset is to extract the sentiments from spoken language information by applying the audio segments and the corresponding transcripts. We used binary labels (positive and negative) based on the sign of the annotations average. We used an 80–20 training-testing split that considers speaker independency. Specifically, there are 1755 utterances for training and 444 utterances for testing.

**IEMOCAP:** The interactive emotional dyadic motion capture database is a multimodal emotion dataset including visual, audio, and text data (Busso et al., 2008). For this study, we only used the audio and text data and classified emotion at the utterance-level. We used the label agreed on by the majority and combined the *happy* and *excited* classes following previous research (Poria et al., 2016). The final dataset consists of four categories including 1591 *hap (happy+excited)*, 1054 *sad*, 1076 *anger*, 1677 *neutral*. We still used an 80–20 speaker independent data split. Table 1 shows the detailed separation.

**MOUD:** The MOUD dataset is a Spanish multimodal utterance-level dataset. Following previous research (Poria et al., 2016), we only consider the positive and negative labels during training and testing. Instead of translating the sentences into English as previous research did, we initialize the word embedding layer randomly.

In addition, we tested the generalizability of the proposed system on a trauma resuscitation speech dataset (TRS).

**TRS:** This dataset was collected from 50 actual trauma cases with 9104 utterance-level audio segments. For each segment, it contains one utterance with at least 2 seconds. The dataset contains the following utterance-level medical category labels: *airway*, *breathing*, *circulation*, *disability*, *exposure*, *secondary-survey*, and *others*. Each utterance was assigned one category by trauma experts. The audio data was collected by two shotgun microphones placed in the resuscitation room. We used two different transcripts as the text input: human transcribed text and speech-to-text transcript. These experiments can then evaluate the influence of noise in the text branch. We reserved 40 cases as the training set and the 10 others as the testing set.

### 4.2 Baselines

We first compared our system with several state-of-the-art methods.

**SVM Trees:** an ensemble of SVM trees was used for classifying concatenated bag-of-words and LLDs (Rozgic et al., 2012).

**BL-SVM:** extracted bag-of-words and low-level descriptors as textual and acoustic features, respectively. The model used an SVM classifier (Rosas et al., 2013).

**GSV-eVector:** this model used Gaussian Supervectors to select LLDs as acoustic features and extracted a set of weighted handcrafted vectors (eVector) as textual features. A linear kernel SVM was used as the final classifier (Jin et al., 2015).

**C-MKL:** the system used a multiple kernel learning structure as the final classifier (Poria et al., 2016). The model extracted textual and acoustic features by using a convolution neural network and OpenS-MILE software, respectively.

**TFN:** a tensor fusion network was used to fuse the extracted features from different modalities (Zadeh et al., 2017).

**WF-LSTM:** a word-level LSTM with temporal attention structure to predict sentiments on the CMUMOSI dataset (Chen et al., 2017).

**BC-LSTM:** a bidirectional LSTM structure to learn contextual information among utterances (Poria et al., 2017b).

**H-DMS:** a hybrid deep multimodal structure to extract and fuse the textual and acoustic features on the IEMOCAP dataset (Gu et al., 2018).

We further tested the performance of models using different feature extraction methods.

**BoW:** using bag-of-words as the textual features to make the final prediction (Wӧllmer et al., 2013).

**WEV:** directly using word embedding vectors as the textual features (Zadeh et al., 2018).

**CNNs-t:** Convolutional neural networks were used for extracting the textual features based on embedding word vectors (Poria et al., 2015).

**LSTM-t:** using an LSTM structure to learn contextual word-level textual features (Gu et al., 2017b).

**OpenSimle:** extracts 6373 low-level acoustic features from an entire audio clip (Poria et al., 2017b).

**COVAREP:** extracts low-level acoustic features including MFCCs, pitch tracking, glottal source parameters, peak slope, and maxima dispersion quotients (Chen et al., 2017).

**CNNs-a:** using convolutional neural networks on extracted MFSCs (Gu et al., 2017).

**LSTM-a:** using an LSTM structure to learn the temporal associations based on LLDs extracted by OpenSmile (Gu et al., 2018).

To make the comparison more reasonable, we introduced a shallow fusion and a deep fusion that combines with the previous feature extraction strategies to make the final predictions.

**SVM:** an SVM was trained on modality-specific features or concatenated features for classification.

**DF:** a deep neural network with three hidden layers was trained as the fusion module and a softmax classifier was used for decision-making.

### 4.3 Implementation

We implemented the system in Keras using the Tensorflow backend. Instead of directly training the entire network, we first pre-trained the feature extraction networks by using two individual softmax classifiers. Then, we tuned the entire network by combining the feature extraction module and modality fusion module. The system was trained on a GTX 1080 GPU with 32GB RAM. We set 256 as the dimension for the bidirectional LSTM. We selected the ReLU activation function except for the attention layers. To overcome overfitting and internal covariate shift (Ioffe and Szegedy, 2015), we applied dropout and batch normalization after the bidirectional LSTM layer and attention layers. We initialized 0.01 as the learning rate, used the Adam optimizer, and binary/categorical cross-entropy loss. We further split 20 percent of the data from the training set as validation and used mini-batch size 8. To make a fair comparison between the proposed system and baselines, we re-trained all models on the same training-testing set split (shown in Table 1). We directly built the models for the baselines that provided the source code. For the rest, we re-implemented the models based on the methods described in their papers.

## 5 Experiment Result

We first compared the performance of the proposed system with the previous methods. The result shows that our system achieves state-of-the-art on all three published datasets. Specifically, we achieved 76.2% accuracy and 74.8 weighted F1 score on CMU-MOSI, outperforming the previous methods by a margin of 2.3% to 7.8%, which demonstrates the effectiveness of the proposed architecture. Compared to the traditional approaches using low-level handcrafted features and shallow fusion strategies (GSV-eVector and SVM Trees), the proposed method shows a significant performance improvement on IEMOCAP (9.3% and 8.7% accuracy gain, respectively). Experiments also indicate that our system performs better than the deep approaches (including C-MKL, TFN, H-DMS), showing the necessity of learning attentive information on feature extraction and fusion levels. Our approach achieves a competitive result (72.8% accuracy) on the MOUD dataset. We further re-implemented all previous methods on the TRS dataset, and our system reports the best performance in terms of both accuracy (69.4%) and weighted F1 score (66.0).

We further compare low-level vs high-level features and shallow vs deep fusion. We re-trained all the individual feature extraction baselines and fusion structures on both IEMOCAP and CMU-MOSI with the same training-testing split. As shown in Table 3 (a), (b), and (c), we made several different combinations of the feature extraction baselines with fusion baselines. We first evaluated the performance of unimodal and multi-modal systems. From Table 3 (a), in all of combinations, multi-modal systems performed better than unimodal ones. In general, the performance of text is similar to that of audio on the IEMOCAP dataset, but text dominates the system performance on MOSI. This might because humans rely more on vocal delivery to express emotions, but less on sentiments. Combining textual and acoustic modalities using an ATFE+AAFE structure leads to 9.6% performance boost on IEMOCAP, which proves the necessity of using multimodal inputs in spoken language understanding. However, there is only 1.7% accuracy improvement on CMU-MOSI by using a multimodal structure. This might because humans express their attitudes without using many vocal characteristics.

Table 3 (b) compares the different feature extraction methods. Compared to traditional textual feature extraction (BoW), the deep models achieve better performance by extracting high-level associations on both datasets. It worth mentioning that directly using the word vectors extracted by *word2vec* model as textual features (WEA+SVM) cannot outperform CNN and LSTM word vector feature extractors (CNNs-t+SVM and LSTM-SVM). This observation demonstrates the necessity of extracting high-level features. On IEMOCAP, the high-level acoustic features extracted by CNNs-a and LSTM-a achieves 59.9% and 60.5% accuracy, outperforming the low-level handcrafted acoustic features (Open-Simle+SVM and COVAREP+SVM) between 1.7% to 7.8% in accuracy. We notice that applying the LSTM architecture over the LLDs gives a 2.4% accuracy increase compared to directly using the LLDs on CMU-MOSI, which shows that modeling the temporal associations improve system performance. As expected, the proposed attention-based textual and acoustic feature extraction performs the best on each individual branch. Based on the above observations, we conclude that learning the high-level features from textual and acoustic data improves the system performance, and that the proposed attention-based LSTM structure indeed helps extract associated features.

**Figure 5:**
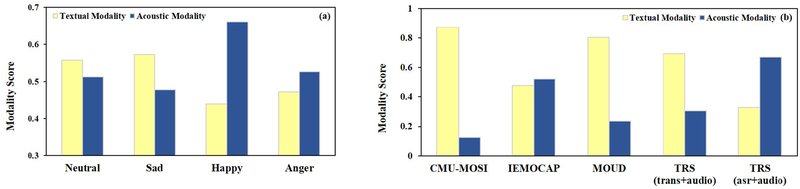
The weighted scores of modality attention. (a) Modality attention scores of different categories on IEMOCAP. (b) Modality attention scores of different datasets.

Compared to the performance of shallow fusion (SVM) in Table 3 (c), deep fusion (DF) gives a significant performance improvement on combinations that use deep feature extractors (CNNs, LSTM, and proposed attention structure), demonstrating that extracting associations across modality-specific features indeed helps the final decision-making. The modality fusion outperforms both shallow fusion (directly using SVM classifier) and deep fusion (DF) on diverse feature extraction combinations. Using an MAF structure instead of SVM and DF brings 5.1% and 1.4% accuracy gain on CMU-MOSI, respectively. To further compare, we visualized the weighed scores from the modality attention on different datasets and categories (shown in Figure 5). We computed the average scores of one hundred random testing samples from each category and dataset. The results indicate the proposed modality attention can learn the distinct scores on different categories and datasets.

We further tested the generalization of the proposed system by applying it to the TRS dataset. Instead of just using the transcribed speech text, we fed the raw audio data into the IBM Watson speech to text API to automatically recognize speech (ASR). From Table 3 (d), using the ASR text leads to a 19.1% accuracy decrease compared to the transcribed text on unimodal systems. However, the multimodal structure only has a 10.5% accuracy drop. These observations indicate that the multimodal system is tolerant to noisy data, demonstrating the generalizability of the proposed multimodal architecture with modality attention.

## 6 Conclusion

In this paper, we introduced a hybrid attention based multimodal architecture for different spoken language understanding tasks. Our system used feature attention and modality attention to select the representative information at both the feature-level and modality-level. The proposed modality attention fusion overcomes the limitations from feature-level and decision-level fusion by performing feature-level fusion with modality scores over the features. We evaluated our system on three published datasets and a trauma resuscitation speech dataset. The results show that the proposed architecture achieves state-of-the-art performance. We also demonstrated the necessity of applying a multimodal structure, extracting high-level feature representations, and using modality attention fusion. The generalization testing established that our system has the ability to handle actual speech data.

## Figures and Tables

**Figure 3:**
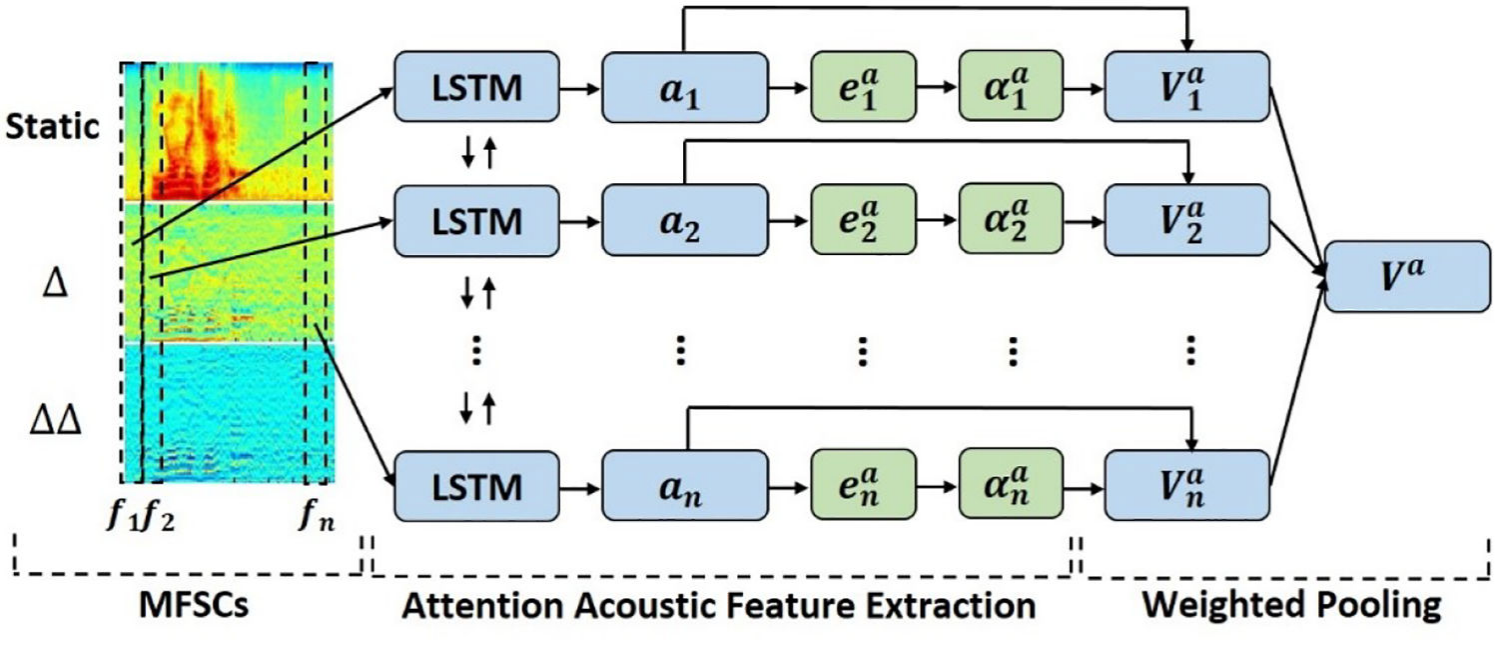
Acoustic feature extraction with attention.

**Table 1:** Dataset details.

Dataset	Class	Speaker Independent	Training Set	Testing Set
CMU-MOSI	2	93 (74|19)	1755	444
IEMOCAP	4	151(121|30)	4295	1103
MOUD	2	79 (59|20)	322	115
TRS	7	50 (40|10)	7261	1843

**Table 2:** Proposed system vs previous methods. Acc = accuracy (%). W-F1 = weighted F1 score.

Approach	CMU-MOSI		IEMOCAP		MOUD		TRS	
	Acc	W-F1	Acc	W-F1	Acc	W-F1	Acc	W-F1
SVM Tree	67.3	66.1	66.4	66.7	60.4	50.4	58.4	45.7
BL-SVM	68.4	67.8	65.2	65.0	60.3	52.8	59.2	50.1
GSV-eVector	65.7	65.5	64.2	64.3	61.1	52.3	58.4	48.4
C-MKL	71.3	71.0	67.0	67.2	72.0	72.2	62.1	58.1
TFN	73.6	73.5	70.4	70.2	62.1	61.2	64.4	61.5
WF-LSTM	73.9	73.3	69.5	69.4	72.7	72.8	65.6	61.5
BC-LSTM	72.4	72.6	70.8	70.8	72.4	72.4	67.9	64.4
H-DMS	70.4	70.2	70.2	69.8	68.4	67.6	66.7	64.3
Our Method	**76.2**	**74.8**	**72.1**	**72.2**	**72.8**	**73.0**	**69.4**	**66.0**

**Table 3:** Detailed comparison on CMU-MOSI (CM) dataset and IEMOCAP (IE) dataset (accuracy percentage). OS* = OpenSmile. COV* = COVAREP. ATFE = proposed attention based textual feature extraction. AAFE = proposed attention based acoustic feature extraction. MAF = modality attention fusion.

**(a) Comparison of modalities**			**(b) Comparison of Features**					
Approach	CM	IE	Approach	CM	IE	Approach	CM	IE
BoW+SVM	65.3	53.2	BoW+SVM	65.3	53.2	OS*+SVM	52.9	56.4
OS*+SVM	52.9	56.4	WEV+SVM	65.4	54.7	COV*+SVM	51.5	52.7
BoW+OS*+SVM	65.9	61.7	CNNt+SVM	67.3	55.2	CNNa+SVM	54.1	55.4
CNNt+DF	69.2	57.8	LSTMt+SVM	68.2	55.7	LSTMa+SVM	56.9	56.1
CNNa+DF	57.3	59.9	ATFE+SVM	72.2	61.0	AAFE+SVM	57.1	59.1
CNNt+CNNa+DF	71.6	64.2	CNNt+DF	69.2	57.8	OS*+DF	56.1	58.7
ATFE+DF	74.5	61.8	LSTMt+DF	71.2	58.2	COV*+DF	55.1	56.3
AAFE+DF	60.4	62.5	LSTMa+DF	58.5	60.5	CNNa+DF	57.3	59.9
ATFE+AAFE+MAF	**76.2**	**72.1**	ATFE+DF	**74.5**	**61.4**	AAFE+DF	**60.4**	**62.5**
**(d) Generalization**			**(c) Comparison of Fusion**					
			Approach	CM	IE	Approach	CM	IE
Approach		TRS	BoW+OS*+SVM	65.9	61.7	CNNt+CNNa+SVM	65.7	63.4
AAFE+DF		56.5	BoW+OS*+DF	67.2	63.2	CNNt+CNNa+DF	71.6	64.2
ATFE(trans)+DF		66.8	BoW+OS*+MAF	68.7	64.7	CNNt+CNNa+MAF	72.9	66.1
ATFE(asr)+DF		47.7	WEA+COV*+SVM	65.8	62.7	ATFE+AAFE+SVM	71.1	65.1
ATFE(trans)+AAFE+DF		69.4	WEA+COV*+DF	67.7	64.1	ATFE+AAFE+DF	74.8	70.5
ATFE(asr)+AAFE+DF		58.9	WEA+COV*+MAF	68.5	64.8	ATFE+AAFE+MAF	**76.2**	**72.1**
